# The association between fatty acid quality indices and quality of life among overweight and obese women: A cross-sectional study

**DOI:** 10.3389/fpubh.2022.1030726

**Published:** 2023-01-26

**Authors:** Niloufar Rasaei, Elnaz Daneshzad, Neda Soveid, Cain C. T. Clark, Khadijeh Mirzaei

**Affiliations:** ^1^Department of Community Nutrition, School of Nutritional Sciences and Dietetics, Tehran University of Medical Sciences (TUMS), Tehran, Iran; ^2^Non-Communicable Diseases Research Center, Alborz University of Medical Sciences, Karaj, Iran; ^3^Centre for Intelligent Healthcare, Coventry University, Coventry, United Kingdom

**Keywords:** fatty acid quality indices, quality of life, overweight, obese, women

## Abstract

**Background:**

Reduced quality of life (QOL) is a major public health challenge affecting the global population. Fatty acid quality indices (FAQIs) are novel determinants of QOL and may impact various aspects of QOL. Prior research has established a significant link between dietary habits and QOL. However, the association between FAQIs and specific dimensions of QOL has not been established. Therefore, we aimed to investigate the association between FAQIs and QOL in overweight and obese women.

**Methods:**

In total, 378 adult overweight or obese women participated in this cross-sectional study. Several anthropometric indices, systolic and diastolic blood pressure, and biochemical factors were measured using standard protocols. Dietary intake was assessed using a validated and reliable semi-quantitative food frequency questionnaire (the FFQ, 147 items). The cholesterol–saturated fat index (CSI) and the ratio of omega-6/omega-3 (N6/N3) essential fatty acids consumed were employed as FAQIs. The SF-36 questionnaire was administered to measure QOL. Linear logistic regression was used, in the form of raw and adjusted models, to evaluate the associations between FAQIs and QOL.

**Results:**

The study sample consisted of 279 participants for whom ω-6/ω-3 ratio was measured and 378 participants for whom CSI was measured. The mean (±SD) age of participants was 36.65 ± 9.07 years. Linear logistic regression, with adjustment for potential confounders, such as age, energy intake, body mass index, employment, and thyroid status, indicated that ω-6/ω-3 intake ratio was negatively and marginally significantly associated with general health (β = −139.94, 95% CI: [−286.54, 6.66]; *p* = 0.061**)** and physical role limitations (β= −337.68, 95% CI: [−679.99, 1.61]; *p* = 0.051). A significant negative association was observed between ω-6/ω-3 intake ratio and social functioning (β = −247.54, 95% CI: [−458.14, −36.94]; *p* = 0.021), which indicates that obese and overweight women with a higher ω-6/ω-3 intake ratio obtained lower scores on social functioning.

**Conclusions:**

It was found that FAQI scores were negatively associated with certain QOL measures among overweight and obese Iranian women, suggesting that a higher consumption of fatty acids, especially trans and saturated fatty acids, may be associated with lower QOL.

## Introduction

According to epidemiological studies, the prevalence of overweight and obesity is rising worldwide (1). Current trends predict that 2.16 billion adults will have a BMI ≥ 25 kg/m^2^ by 2030 (2). The prevalence of obesity has been reported to be 76.4% in developing countries, such as Iran, and this level of incidence has also been found to be associated with a lower quality of life and early death (3). Quality of life (QOL) is defined by the World Health Organization (WHO) as a person's perception of their life, based on their culture and value system, as well as their goals, expectations, standards, and concerns (4). QOL refers to an individual's perception of his or her current state of health, based on physical and social function, physical and emotional limitations on the ability to carry out roles, vitality, bodily pain, and mental and general health (5–7). There are objective and subjective components to QOL, each of which are multidimensional and dynamic (8). Compared to men, women appear to have lower QOL (9); in addition to this gender difference, obese and non-obese people have differing QOL scores (10). QOL can be affected by nutritional factors such as dietary intake (6), and research has long suggested that quality of life may be associated with diet quality (11); therefore, a number of eating indices have been developed to assess the quality of an individual's diet. For instance, in a cross-sectional study, it was found that students with a higher quality diet and normal body weight are significantly more likely to have higher quality of life scores than students who consume a less healthy diet and are overweight or obese (12).

Fats are an important part of the diet and the primary source of energy (13). Previous research has concentrated on quantity of fat intake; however, according to contemporary research, the quality of dietary fat may also have a major impact on health status and, as a consequence, quality of life (14, 15). In this regard, Connor et al. (16) have developed a new tool for measurement of dietary fat quality, known as the Cholesterol–Saturated Fat Index (CSI). In addition to the CSI, Simopoulos has emphasized the importance of the ratio of omega-6 to omega-3 (N6/N3) essential fatty acids (EFAs) (17). Thus, the prevention and management of chronic diseases may be strongly linked to the maintenance of a balanced N6/N3 EFA ratio (17). Dietary self-monitoring tools, such as the CSI, allow patients to better understand the cholesterol and saturated fatty acid (SFA) content of food, enabling them to decrease their dietary cholesterol and SFA intake through better management and food choices. Indeed, consumption of foods with lower CSI scores signifies reduced SFA and cholesterol intake (18). Previous work has suggested that high levels of dietary SFA might contribute to physical dysfunction and general health disturbances; additionally, trans fatty acid (TFA) is associated with mental disorders (social function, emotional role limitations, and vitality) and bodily pain (19). Furthermore, previous studies have shown that QOL may be improved following an increase in intake of omega-3 fatty acids *via* dietary adjustment or supplementation (20, 21). In support of this hypothesis, individuals adhering to a western dietary pattern, which is high in SFA and low in omega-3 EFA, have been found to exhibit a lower risk of low QOL (20). However, some studies have indicated that oral supplementation of omega-3 polyunsaturated fatty acids (PUFAs) does not affect QOL (22–24).

Given the conflicting nature of these findings, and because no existing study has assessed FAQIs along with QOL, especially in high-risk groups such as overweight and obese women, we aimed to evaluate the association between FAQIs and QOL among obese and overweight women. Furthermore, to the authors' knowledge, there is no currently available literature on the association between fatty acid quality indices (FAQIs) and QOL. Specifically, most potentially relevant work has only evaluated a single specific type of fat (20, 22) or total fat in the diet as a whole (25, 26), rather than using two comprehensive indices. This demonstrates the importance of conducting further research in this field.

## Methods

### Study population

Overweight and obese women (*N* = 378) who were referred to health centers in Tehran, Iran were recruited to participate in the present cross-sectional study. All participating individuals provided a signed declaration of their written informed consent at the beginning of the study. Individuals falling within an age range of 18–68 years and a BMI range of 25–40 kg/m^2^ were eligible for inclusion, while the exclusion criteria were as follows: pregnancy or menopause; lactation; smoking; dieting during the past year; weight loss supplementation; use of antipsychotic, antihypertensive, or glucose- or lipid-lowering medications; malignancies; depression; all types of diabetes; liver, kidney, or cardiovascular diseases; and any other acute or chronic diseases. The Ethics Committee of Tehran University of Medical Sciences (TUMS) approved the present study (assigned approval number: IR.TUMS.VCR.REC.1399.636).

In this cross-sectional study, overweight and obese women were recruited from 20 health centers in all regions of West and Central Tehran, using community-based multi-stage simple random sampling. The 20 health centers were themselves randomly selected from all health centers affiliated with Tehran University of Medical Sciences. Sampling was such that individuals who were referred to the Tehran health centers and who met the inclusion criteria were randomly selected for enrollment in the study.

### Anthropometrics and blood pressure assessment

A bioelectrical impedance analyzer (BIA; InBody 770 scanner from InBody Co., Seoul, Korea) was used to take anthropometric measurements, including weight, body mass index (BMI), body free mass (BFM), bone mineral content (BMC), visceral fat area (VFA), fat-free mass (FFM), fat-free mass index (FFMI), fat mass index (FMI) and body fat percent (BF%), in accordance with the manufacturer's protocols (27). Participants were requested to remove extra clothing and metal objects, such as watches, rings, earrings, shoes, sweaters, and coats, prior to having these measurements taken. Height was measured to within 0.5 cm precision using a non-stretch tape measure with participants in a standing position and barefoot. Similarly, waist circumference (WC) and hip circumference (HC) were measured to within 0.5 cm precision using a non-stretch tape measure at the narrowest section of the waist and the widest part of the buttocks, respectively. Waist-to-hip ratio (WHR) was calculated by dividing WC by HC. Finally, blood pressure was measured twice after 5 min of rest using an appropriate cuff for each participant's arm size. The average of the two measurements is reported.

### Biochemical and hormonal measures

Venous blood was collected after participants had fasted overnight. Serum samples were stored at −80 °C after centrifuging. Standard methods were used to assess all samples at the Nutrition and Biochemistry Laboratory of the School of Nutritional Sciences and Dietetics at TUMS. Fasting blood glucose (FBS), triglyceride (TG), and total cholesterol (TC) were measured using glucose oxidase–phenol 4-aminoantipyrine peroxidase (GOD-PAP) and glycerol-3-phosphate oxidase–phenol 4-aminoantipyrine peroxidase (GPOPAP) enzymatic endpoints, respectively. We measured low-density lipoprotein (LDL) and high-density lipoprotein (HDL) cholesterol using direct enzymatic clearance assay. The minimum detectable concentration of insulin was 1.76 mIU/mL, and the intra-assay coefficient of variation (CV) and inter-assay CV were 2.19% and 4.4%, respectively. HOMA-IR was calculated using the formula: (fasting plasma glucose × fasting serum insulin)/22.5 (28). High-sensitivity C-reactive protein (Hs-CRP) was measured *via* standard protocols. A Randox Laboratories (Hitachi 902) kit was used for all measurements. Finally, the enzymatic endpoint method was used to measure liver enzymes, including serum glutamic oxaloacetic transaminase (GOT) and serum glutamic pyruvic transaminase (GPT).

### Quality of life

We used an instrument known as the MOS 36-item Short Form Health Survey (SF-36) to measure QOL. This self-administered questionnaire contains 36 questions, 35 of which fall into eight multi-item subscales covering physical functioning (PF), physical role limitations (PR), bodily pain (BP), general health (GH), vitality (VT), social functioning (SF), emotional role limitations (ER), and mental health (MH). PF is a 10-question subscale that captures the respondent's ability to deal with the physical requirements of life, such as attending to personal needs, walking, and flexibility. PR is a 4-item subscale that assesses the extent to which the respondent's physical capabilities limit their activity. BP is a 2-item subscale that assesses the perceived amount of pain experienced during the most recent 4 weeks and the extent to which that pain has restricted the respondent's normal work activities. GH is a 5-item subscale that assesses general health in terms of the respondent's personal perceptions. VT is a 4-item subscale that assesses feelings of pep, energy, and fatigue. SF is a 2-item subscale that assesses the extent to which and frequency with which the respondent's physical health or emotional problems have interfered with social interactions with family, friends, and others during the most recent 4 weeks, if at all. ER is a 3-item subscale that assesses the extent, if any, to which emotional factors have restricted the respondent's work or other activities. Finally, MH is a 5-item subscale that evaluates feelings, principally those relating to anxiety and depression. The SF-36 also includes a single question that asks the respondent to provide a self-evaluation of their health changes in the past year (reported health); this question does not fall into any of the eight dimensions and the response is not included in calculating the total SF-36 score. Each of the dimensions mentioned produces a score between 0 (lowest QOL) and 100 (highest QOL) (6, 7, 29).

### Dietary intake assessment

A validated and reliable 147-item semi-quantitative food frequency questionnaire (FFQ) was used to collect dietary intake information (30). All participants recorded their usual frequency of consumption of food items throughout a day, week, or month over the last year. All FFQs were completed in the presence of an expert dietitian. Dietary intake was analyzed for energy intake, macronutrients, and micronutrients (gr/day) using the NUTRITIONIST 4 food analyzer (First Data Bank, San Bruno, CA) (31).

### Fatty acid quality indices

Fatty acid quality was defined on the basis of two indices: the Cholesterol–Saturated Fat Index (CSI) and the ratio of N6 to N3 essential fatty acids. The CSI measures the concentrations of cholesterol and saturated fat in foods; this index was developed by dividing the cholesterol content of a food item by the saturated fat content (18). A lower CSI represents lower cholesterol and/or saturated fat content, which means that a diet consisting of foods with lower CSI has hypocholesterolemic and low atherogenic potential. The ω-6/ω-3 ratio for food items assessed by the FFQ was measured by dividing ω-6 content by ω-3 content (16, 32).

### Assessment of physical activity and other details

Physical activity (PA) was estimated based on a validated and reliable self-report questionnaire, namely the short form of the International Physical Activity Questionnaire (IPAQ). The reliability and validity of the IPAQ have already been assessed in Iranian adults. We measured participants' PA levels during the preceding week and report these in the form of metabolic equivalent (MET) (33). Scores were calculated according to the frequency of engaging in and time spent on light, moderate, high-intensity, and very high-intensity activities, based on a list of common daily activities. Additionally, several variables were assessed using a standard self-report sociodemographic questionnaire: employment (housekeeper, laborer, management employee, non-managerial employee, household worker, or university student), level of education (illiterate, primary education, intermediate education, high school education, diploma, bachelor's degree or higher, or postgraduate education), marital status (married, single, separated from spouse for more than 6 months, widowed, or divorced), economic status (very low income, low income, moderate income, or high income), and intake of supplements.

### Statistical analyses

The Kolmogorov–Smirnov test was conducted to evaluate the normality of distribution of the data. The general characteristics of the obese and overweight women who participated are reported in the form mean ± standard deviation (SD) by N6/N3 intake tertile or CSI tertile. Analysis of covariance (ANCOVA) was used to investigate scores on QOL subscales among each N6/N3 or CSI tertile with adjustment for age, BMI, physical activity, and total energy intake. ANCOVA was also used to investigate differences in dietary intake among tertiles of N6/N3 and CSI with adjustment for total energy intake. Linear logistic regression, including raw and adjusted models, was used to evaluate the associations between FAQI and QOL. Adjustments were made for age, energy intake, BMI, employment, and thyroid status. All statistical analyses were carried out using SPSS version 23.0 (SPSS, Chicago, IL, USA). A *P-*value lower than 0.05 was considered to represent statistical significance, and a *P*-value lower than 0.1 was considered to represent marginal significance.

## Results

### Study population characteristics

In total, the participant sample in the present study consisted of 279 participants for whom N6/N3 ratio was measured and 378 participants for whom CSI was measured. The mean (SD) age of participants was 36.65 (9.07) years, and the mean (SD) BMI was 31.03 (3.87) kg/m^2^. Seventy percent of the participants were married, and 57% were housekeepers. Approximately 47% of participants used supplements. Forty percent of the subjects fell into the moderate income bracket, and 47.6% possessed a bachelor's or higher degree. The mean (SD) total QOL score was 61.69 (29.12), and the mean (SD) FAQI estimate for the CSI group and the N6/N3 ratio group was 13.24 (5.71) per day and 12.65 (0.10) mg/day, respectively.

### Sample characteristics by N6/N3 and CSI tertile

Table 1 shows the general characteristics of the study population by N6/N3 or CSI tertile. In this table, the *p*-values associated with each variable are reported in two forms: raw, and adjusted for age, BMI, physical activity, and total energy intake. In the raw model there was a statistically significant difference in age among participants in different CSI tertiles (*p* = 0.018). In the adjusted model, after controlling for potential confounders, the inter-tertile differences in education and economic status became significant (*p* = 0.009).

**Table 1:** General sample characteristics of obese and overweight women by N6/N3 intake ratio tertile (*n* = 279) or CSI tertile (*n* = 378).

**Variables^†^**	**N6/N3**				
	**Mean** ± **SD**			***P*** **-value**	***P*** **-value^b^**
	**T_1_ (*n =* 93)**	**T_2_ (*n =* 93)**	**T_3_ (*n =* 93)**		
Age (years)	35.95 ± 8.20	36.08 ± 8.45	37.40 ± 8.72	0.434	0.294
PA (MET-min/week)	960.3684 ± 926.0764	1,192.299 ± 1,445.859	812.7500 ± 727.6088	0.082	0.148
**Anthropometric measurements**					
Weight (kg)	81.12 ± 10.74	80.84 ± 11.89	78.01 ± 9.77	0.098	0.788
Height (cm)	162.02 ± 5.47	161.79 ± 5.77	160.15 ± 6.09	0.058	0.655
WC (cm)	98.81 ± 9.13	99.62 ± 10.11	96.79 ± 8.49	0.103	0.238
WHR	0.92 ± 0.47	0.94 ± 0.55	0.92 ± 0.49	0.075	0.263
BMI (kg/m^2^)	30.90 ± 3.93	30.91 ± 3.63	30.37 ± 3.61	0.532	0.462
BF (%)	41.20 ± 5.88	41.05 ± 5.15	41.55 ± 4.91	0.809	0.464
VFA (cm^2^)	162.88 ± 37.06	161.11 ± 40.92	148.08 ± 33.64	0.677	0.766
FFMI	17.91 ± 1.35	19.47 ± 13.52	17.63 ± 1.41	0.236	0.514
FMI	13.02 ± 3.14	12.86 ± 2.86	12.84 ± 2.97	0.902	0.216
FFM (kg)	47.07 ± 4.96	47.48 ± 5.84	45.22 ± 5.07	0.009	0.189
BFM (kg)	33.99 ± 7.88	33.73 ± 7.99	32.42 ± 6.86	0.324	0.550
BMC	2.70 ± 0.32	2.71 ± 0.38	2.57 ± 0.30	0.12	0.327
**Blood pressure**					
SBP (mmHg)	110.35 ± 14.18	112.51 ± 12.88	110.59 ± 13.55	0.503	0.294
DBP (mmHg)	76.94 ± 10.37	78.08 ± 9.42	77.62 ± 9.10	0.727	0.270
**Biochemical variables**					
FBS (mg/dl)	87.06 ± 9.31	86.35 ± 9.12	88.22 ± 10.43	0.468	0.055
TC (mg/dl)	178.53 ± 29.15	184.55 ± 37.64	187.71 ± 39.35	0.260	0.103
TG (mg/dl)	118.33 ± 67.37	121.08 ± 72.83	123.72 ± 70.10	0.119	0.240
HDL (mg/dl)	46.18 ± 10.16	47.51 ± 11.04	46.42 ± 10.70	0.335	0.810
LDL (mg/dl)	92.81 ± 20.49	94.91 ± 24.87	94.98 ± 25.90	0.813	0.853
GOT (u/l)	18.19 ± 8.94	18.31 ± 6.49	16.95 ± 6.41	0.431	0.628
GPT (u/l)	19.76 ± 16.15	19.81 ± 12.29	17.76 ± 10.08	0.521	0.612
Insulin (mIU/mL)	1.21 ± 0.24	1.23 ± 0.22	1.19 ± 0.21	0.537	0.324
HOMA index	3.22 ± 1.27	3.23 ± 1.27	3.54 ± 1.30	0.198	0.033
hs.CRP (mg/l)	4.83 ± 4.48	5.12 ± 5.02	4.36 ± 4.57	0.543	0.373
**Education, % (** ***n*** **)**					
Illiterate	0.0 (0)	2.2 (2)	1.1 (1)	0.608	0.462
Primary education	4.3 (4)	7.5 (7)	2.2 (2)		
Intermediate education	6.5 (6)	4.3 (4)	7.5 (7)		
High school education	2.2 (2)	3.2 (3)	2.2 (2)		
Diploma	32.3 (30)	28.0 (26)	26.9 (25)		
Bachelor's degree or higher	4.3 (4)	10.8 (10)	10.8 (10)		
Postgraduate education	49.5 (46)	44.1 (41)	48.4 (45)
**Employment, % (** ***n*** **)**					
Housekeeper	62.4 (58)	61.3 (57)	52.7 (49)	0.291	0.215
Laborer	1.1 (1)	2.2 (2)	0.0 (0)
Management employee	9.7 (9)	17.2 (16)	22.6 (21)
Non-managerial employee	15.1 (14)	12.5 (2)	37.5 (6)
Household worker	1.1 (1)	2.2 (2)	3.2 (3)
University student	7.5 (7)	6.5 (6)	5.4 (5)
**Marriage, % (** ***n*** **)**					
Married	75.3 (70)	78.5 (73)	77.4 (72)	0.575	0.614
Single	19.4 (18)	19.4 (18)	19.4 (18)
Separated from spouse for more than 6 months	0.0 (0)	0.0 (0)	1.1 (1)
Widowed	2.2 (2)	0.0 (0)	0.0 (0)
Divorced	2.2 (2)	2.2 (2)	1.1 (1)
**Economic status, % (** ***n*** **)**					
Very low income	2.6 (2)	1.3 (1)	2.8 (2)	0.872	0.846
Low income	3.9 (3)	4.0 (3)	6.9 (5)
Moderate income	34.2 (26)	41.3 (2)	40.3 (29)
High income	59.2(45)	53.3(40)	50.0 (36)
**Supplementation, % (** ***n*** **)**					
Yes	59.7 (46)	62.3 (48)	51.4 (37)	0.371	0.249
No	40.3 (31)	37.7 (29)	48.6 (35)		
**Variables** ^†^	**CSI**				
	**Mean** ± **SD**			***P*** **-value**	***P*** **-value** ^*b*^
	**T**_1_ **(*****n** =* **126)**	**T**_2_ **(*****n** =* **126)**	**T**_3_ **(*****n** =* **126)**		
Age (years)	37.87 ± 8.95	37.34 ± 9.22	34.81 ± 9.19	**0.018**	0.473
PA (MET-min/week)	839.3274 ± 1027.524	1,092.786 ± 1,205.068	1,048.355 ± 1,046.475	0.181	0.366
**Anthropometric measurements**					
Weight (kg)	79.79 ± 10.58	80.12 ± 11.20	81.95 ± 12.10	0.265	0.868
Height (cm)	160.68 ± 5.86	161.27 ± 5.86	161.64 ± 5.63	0.417	0.963
WC (cm)	98.62 ± 9.09	98.73 ± 9.52	100.19 ± 10.09	0.348	0.841
WHR	0.93 ± 0.04	0.94 ± 0.05	0.93 ± 0.05	0.555	0.787
BMI (kg/)	30.95 ± 3.70	30.76 ± 3.77	31.33 ± 4.07	0.495	0.553
BF (%)	42.03 ± 5.27	41.60 ± 4.95	42.48 ± 5.80	0.427	0.628
VFA (cm^2^)	163.06 ± 37.75	163.45 ± 36.85	170.32 ± 37.99	0.226	0.514
FFMI	17.82 ± 1.48	18.88 ± 11.70	17.77 ± 1.54	0.351	0.328
FMI	13.25 ± 3.12	12.96 ± 2.49	13.55 ± 3.25	0.331	0.262
FFM (kg)	46.06 ± 5.34	46.48 ± 5.43	46.53 ± 5.77	0.758	0.558
BFM (kg)	33.88 ± 7.79	33.59 ± 7.60	35.32 ± 8.55	0.184	0.589
BMC	2.61 ± 0.32	2.66 ± 0.36	2.66 ± 0.35	0.431	0.554
**Blood pressure**					
SBP (mmHg)	112.88 ± 13.58	111.01 ± 14.02	109.54 ± 12.71	0.280	0.708
DBP (mmHg)	78.52 ± 9.84	77.66 ± 8.49	76.24 ± 10.63	0.314	0.646
**Metabolic factors**					
FBS (mg/dl)	87.98 ± 10.60	87.14 ± 9.70	86.41 ± 8.23	0.621	0.992
TC (mg/dl)	184.75 ± 31.24	184.22 ± 39.71	181.56 ± 36.17	0.857	0.661
TG (mg/dl)	123.69 ± 81.12	124.06 ± 70.69	113.43 ± 49.64	0.602	0.701
HDL (mg/dl)	47.40 ± 10.41	46.42 ± 12.13	46.09 ± 8.42	0.732	0.874
LDL (mg/dl)	95.08 ± 23.11	92.84 ± 25.15	95.16 ± 23.15	0.779	0.308
GOT (u/l)	16.94 ± 6.28	18.07 ± 7.72	18.53 ± 8.12	0.393	0.447
GPT (u/l)	19.76 ± 16.15	19.81 ± 12.29	17.76 ± 10.08	0.820	0.815
Insulin (mIU/mL)	1.19 ± 0.23	1.22 ± 0.21	1.12 ± 0.23	0.684	0.580
HOMA index	3.42 ± 1.40	3.17 ± 1.17	3.48 ± 1.27	0.269	0.422
hs.CRP (mg/l)	4.58 ± 4.28	5.05 ± 4.75	5.56 ± 4.63	0.230	0.373
**Education, % (** ***n*** **)**					
Illiterate	2.4 (3)	0.8 (1)	0.0 (0)	0.166	**0.009**
Primary education	4.8 (6)	3.2 (4)	2.4 (3)		
Intermediate education	10.3 (13)	4.8 (6)	4.8 (6)		
High school education	3.2 (4)	0.8 (1)	2.4 (3)		
Diploma	28.6 (36)	35.7 (45)	28.6 (36)		
Bachelor's degree or higher	9.5 (12)	7.1 (9)	4.8 (6)		
Postgraduate education	40.5 (51)	46.8 (59)	57.1 (72)
**Employment, % (** ***n*** **)**					
Housekeeper	57.9 (73)	58.7 (74)	55.6 (70)	0.575	0.529
Laborer	0.8 (1)	2.4 (3)	0.0 (0)		
Management employee	18.3 (23)	16.7 (21)	16.7 (21)		
Non-managerial employee	12.7 (16)	12.7 (16)	15.9 (20)		
Household worker	6.3 (8)	1.6 (2)	4.8 (6)		
University student	3.2 (4)	6.3 (8)	4.8 (6)
**Marriage, % (** ***n*** **)**					
Married	74.6 (94)	74.6 (94)	65.1 (82)	0.257	0.275
Single	21.4 (27)	20.6 (26)	29.4 (37)		
Separated from spouse for more than 6 months	0.8 (1)	0.8 (1)	0.0 (0)		
Widowed	0.0 (0)	0.0 (0)	2.4 (3)		
Divorce	2.4 (3)	3.2 (4)	3.2 (4)
**Economic status, % (** ***n*** **)**					
Very low income	1.8(2)	4.1(4)	0.0(0)	0.58	**0.009**
Low income	7.3(8)	2.0(2)	3.7(4)		
Moderate income	37.3(41)	37.8(37)	27.8(30)		
High income	53.6(59)	56.1(55)	68.5(74)		
**Supplementation, % (** ***n*** **)**					
Yes	42.0 (47)	55.3 (57)	46.4 (51)	0.138	0.142
No	58.0 (65)	44.7 (46)	53.6 (59)		

BF%, body fat percentage; BFM, body fat mass; BMC, bone mineral content; BMI, body mass index; CSI, cholesterol–saturated fat index; N6/N3, Σ of Omega 6 series/Σ of Omega 3 series; DBP, diastolic blood pressure; FBS, fasting blood sugar; FFM, fat-free mass; FFMI, fat-free mass index; FMI, fat mass index; GOT, Glutamate oxaloacetate transaminase; GPT, glutamate pyruvate transaminase; HDL, high-density lipoprotein; HOMA, homeostatic model assessment; hs-CRP, high-sensitivity C-reactive protein; PA, physical activity; SD, standard deviation; SBP, systolic blood pressure; T, tertile; TC, total cholesterol; TG, triglyceride; VFA, visceral fat area; WC, waist circumference.

† Analyzed using analysis of variance (ANOVA).

b Adjusted for age, BMI, physical activity, and total energy intake.

p < 0.05 was considered to represent significance.

### Differences in quality of life among N6/N3 and CSI tertiles

The relationships of N6/N3 ratio and CSI tertiles with total QOL score and QOL component scores are presented in Table 2. For the N6/N3 ratio group, in the raw model, there was a statistically significant difference between the tertiles in terms of total QOL score (*p* = 0.01). In the adjusted model, after controlling for confounders (age, BMI, physical activity, and total energy intake), there was a significant difference between the tertiles on the Bodily Pain subscale (*p* = 0.024) and a marginally significant difference on the emotional role limitations subscale (*p* = 0.056). For the CSI group, in the raw model, there was a statistically significant difference between the tertiles on the Bodily Pain subscale (*p* = 0.045), and a marginally significant difference on two other items, namely physical role limitations (*p* = 0.058) and total quality of life score (*p* = 0.057). In the adjusted model, total quality of life score was the only significant difference among the CSI tertiles (*p* = 0.009).

**Table 2:** QOL scores of overweight and obese women by N6/N3 intake ratio tertile (*n* = 279) or CSI tertile (*n* = 378).

**Variables^†^**	**N6/N3**				
	**Mean** ± **SD**			***P*** **-value**	***P*** **-value^b^**
**SF-36 subscales**	**T**_1_ **(*****n** =* **93)**	**T**_2_ **(*****n** =* **93)**	**T**_3_ **(*****n** =* **93)**		
General Health (SF.1)	66.62 ± 16.14	67.14 ± 15.73	65.93 ± 17.01	0.912	0.327
Physical Functioning (SF.2)	81.500 ± 18.24	81.56 ± 15.79	82.06 ± 14.96	0.967	0.646
Physical Role Limitations (SF.3)	79.68 ± 40.55	85.87 ± 33.96	82.85 ± 37.96	0.652	0.922
Emotional Role Limitations (SF.4)	85.93 ± 35.03	74.60 ± 43.87	71.28 ± 45.42	0.111	**0.056**
Social Functioning (SF.5)	73.39 ± 22.36	74.20 ± 22.34	69.57 ± 25.19	0.471	0.402
Bodily Pain (SF.6)	60.85 ± 21.59	63.22 ± 19.31	55.47 ± 20.62	0.084	**0.024**
Vitality (SF.7)	66.62 ± 19.42	69.48 ± 17.70	66.24 ± 18.28	0.554	0.208
Mental Health (SF.8)	76.95 ± 22.18	78.01 ± 19.75	72.35 ± 24.85	0.300	0.299
Health Transition Item (SF.9)	44.92 ± 25.26	47.22 ± 26.98	44.64 ± 28.19	0.836	0.181
Overall Quality of Life (total SF-36 score)	54.53 ± 31.00	59.63 ± 27.95	69.43 ± 27.06	0.01	0.725
**Variables** ^†^	**CSI**				
	**Mean** ± **SD**			***P*** **-value**	***P*** **-value** ^b^
	**T**_1_ **(*****n** =* **126)**	**T**_2_ **(*****n** =* **126)**	**T**_3_ **(*****n** =* **126)**		
General Health (SF.1)	66.23 ± 16.58	66.82 ± 19.05	66.40 ± 16.46	0.972	0.850
Physical Functioning (SF.2)	82.62 ± 16.32	81.02 ± 17.25	81.84 ± 17.86	0.819	0.470
Physical Role Limitations (SF.3)	76.31 ± 42.42	89.55 ± 30.31	82.10 ± 38.53	**0.058**	0.340
Emotional Role Limitations (SF.4)	69.36 ± 46.23	80.00 ± 40.22	73.46 ± 44.16	0.251	0.691
Social Functioning (SF.5)	69.57 ± 23.62	74.38 ± 24.66	71.57 ± 22.10	0.378	0.504
Bodily Pain (SF.6)	57.61 ± 21.15	64.11 ± 18.35	64.29 ± 22.79	**0.045**	0.139
Vitality (SF.7)	64.47 ± 19.04	68.40 ± 17.72	70.52 ± 20.22	0.528	0.288
Mental Health (SF.8)	73.22 ± 23.01	76.47 ± 22.95	73.94 ± 24.52	0.618	0.755
Health Transition Item (SF.9)	43.25 ± 28.81	48.33 ± 25.63	45.21 ± 25.99	0.422	0.595
Overall Quality of Life (total SF-36 score)	58.45 ± 30.22	67.04 ± 27.63	55.52 ± 29.53	**0.057**	**0.009**

QOL, Quality of Life; CSI, cholesterol–saturated fat index; N6/N3, Σ of Omega 6 series/Σ of Omega 3 series; T, tertile; SF-36, 36-item Short Form Health Survey.

† Analyzed using analysis of covariance (ANCOVA).

b Adjusted for age; BMI, physical activity, and total energy intake.

p < 0.05 was considered to represent significance.

### Differences in dietary intake between N6/N3 and CSI tertiles

The dietary intakes of the sample by N6/N3 and CSI tertile are presented in Table 3. The results show that, in the raw model, there was a significant difference between the N6/N3 tertiles in terms of intake of all food groups and nutrients. After controlling for the potential confounding factor of energy intake, the difference in intake of carbohydrates (*p* = 0.009) and red meat (*p* = 0.058) remained significant and marginally significant, respectively. Among the CSI group, in the raw model, there were statistically significant differences between the tertiles in intake of all food groups except legumes (*p* = 0.166) and all nutrients except vitamin E (*p* = 0.291). After adjusting for energy intake, the difference in intake of legumes remained non-significant (*p* = 0.212), as in the raw model.

**Table 3:** Dietary intake among obese and overweight women by N6/N3 intake ratio tertile (*n* = 279) or CSI tertile (*n* = 378).

**Variables^†^**	**N6/N3**				
	**Mean** ± **SD**			***P*** **-value**	***P*** **-value^b^**
	**T_1_ (*n =* 93)**	**T_2_ (*n =* 93)**	**T_3_ (*n =* 93)**		
**Food group**					
Whole grains (g/d)	76.88 ± 67.78	70.52 ± 59.97	41.42 ± 38.36	**0.00**	0.175
Refined grains (g/d)	489.62 ± 239.45	340.17 ± 194.30	272.29 ± 117.29	**0.00**	0.462
Vegetables (g/d)	439.80 ± 243.54	417.86 ± 256.50	289.23 ± 183.76	**0.00**	0.060
Fruits (g/d)	750.11 ± 382.63	439.53 ± 243.73	325.48 ± 209.00	**0.00**	0.46
Nuts (g/d)	21.11 ± 19.00	15.81 ± 17.75	6.95 ± 6.01	**0.00**	0.369
Legumes (g/d)	51.82 ± 40.69	52.32 ± 44.80	36.50 ± 31.08	**0.008**	0.189
High-fat dairy (ml/d)	142.61 ± 178.93	85.27 ± 120.21	34.16 ± 55.29	**0.00**	0.290
Low-fat dairy (ml/d)	371.77 ± 281.11	299.61 ± 205.06	232.10 ± 152.00	**0.00**	0.818
Eggs (g/d)	25.27 ± 17.06	22.53 ± 13.63	17.38 ± 10.67	**0.001**	0.385
Poultry (g/d)	45.60 ± 55.96	31.70 ± 29.99	28.12 ± 23.10	**0.006**	0.328
Fish (g/d)	13.75 ± 15.65	11.24 ± 11.07	9.36 ± 8.81	**0.049**	0.996
Fast food (g/d)	27.02 ± 34.16	17.00 ± 20.52	13.62 ± 16.33	**0.001**	0.710
Red meat (g/d)	31.64 ± 20.16	20.75 ± 19.16	12.47 ± 8.39	**0.00**	**0.058**
**Nutrient intake**					
Energy (kcal/d)	3,468.728 ± 402.6743	2,545.521 ± 1,903,653	1,799.816 ± 271.0121	**0.00**	-
Protein (g/d)	114.98 ± 24.09	87.51 ± 17.49	62.37 ± 13.30	**0.00**	0.584
Carbohydrate (g/d)	502.95 ± 82.83	353.96 ± 47.13	255.92 ± 53.31	**0.00**	**0.009**
Total fat (g/d)	122.50 ± 27.88	95.28 ± 20.53	63.74 ± 15.19	**0.00**	0.096
Vitamin A (RAE)	988.76 ± 431.13	753.80 ± 405.17	569.09 ± 260.68	**0.00**	0.921
Vitamin D (μg/day)	2.55 ± 2.08	1.91 ± 1.41	1.43 ± 1.03	**0.00**	0.983
Vitamin E (mg/day)	20.03 ± 9.20	18.25 ± 9.65	13.39 ± 7.21	**0.018**	0.212
Vitamin K (mg/day)	244.15 ± 172.96	244.31 ± 264.84	166.70 ± 102.72	**0.00**	0.493
Thiamin (mg/day)	2.71 ± 0.49	2.03 ± 0.38	1.470.32	**0.00**	0.960
Riboflavin (mg)	2.90 ± 0.78	2.1 ± 0.53	1.53 ± 0.37	**0.00**	0.516
Niacin (mg)	33.23 ± 9.4	24.09 ± 5.1	18.06 ± 4.17	**0.00**	0.467
Biotin (μg/day)	48.54 ± 19.21	39.26 ± 12.90	26.85 ± 9.34	**0.00**	0.206
Pantothenic acid (mg/day)	8.48 ± 2.59	6.29 ± 1.36	4.60 ± 1.06	**0.00**	0.399
Vitamin B6 (mg/day)	2.82 ± 0.58	2.10 ± 0.45	1.53 ± 0.34	**0.00**	0.239
Folic acid (μg/day)	866.26 ± 185.96	650.32 ± 145.73	507.57 ± 141.16	**0.00**	0.580
Vitamin B12 (μg/day)	5.85 ± 3.02	4.03 ± 1.71	3.16 ± 1.38	**0.00**	0.452
**Variables** ^†^	**CSI**				
	**Mean** ± **SD**			***P*** **-value**	***P*** **-value** ^b^
	**T**_1_ **(*****n** =* **126)**	**T**_2_ **(*****n** =* **126)**	**T**_3_ **(*****n** =* **126)**		
**Food group**					
Whole grains (g/d)	60.65 ± 59.95	64.81 ± 57.51	94.32 ± 101.21	**0.001**	0.346
Refined grains (g/d)	329.03 ± 210.11	375.64 ± 188.06	386.16 ± 207.17	**0.059**	**0.001**
Vegetables (g/d)	282.37 ± 190.02	410.58 ± 251.52	433.96 ± 245.43	**0.00**	**0.003**
Fruits (g/d)	359.96 ± 288.70	471.84 ± 323.43	518.56 ± 321.90	**0.00**	0.355
Nuts (g/d)	10.15 ± 12.26	15.12 ± 20.69	20.78 ± 21.41	**0.00**	0.741
Legumes (g/d)	40.35 ± 33.72	49.66 ± 42.61	45.80 ± 40.44	0.166	0.212
High-fat dairy (ml/d)	49.50 ± 71.59	76.93 ± 116.12	150.43 ± 189.29	**0.00**	**0.001**
Low-fat dairy (ml/d)	200.60 ± 136.87	310.65 ± 224.68	367.16 ± 249.14	**0.00**	**0.001**
Eggs (g/d)	12.59 ± 7.10	22.17 ± 9.49	38.60 ± 22.42	**0.00**	**0.00**
Poultry (g/d)	21.89 ± 17.23	33.70 ± 25.36	52.21 ± 54.94	**0.00**	**0.00**
Fish (g/d)	7.06 ± 6.41	11.85 ± 11.74	14.10 ± 15.56	**0.00**	**0.001**
Fast food (g/d)	14.48 ± 16.92	15.50 ± 18.41	32.47 ± 37.43	**0.00**	**0.001**
Red meat (g/d)	12.09 ± 8.28	20.95 ± 15.96	32.14 ± 27.39	**0.00**	**0.00**
**Nutrient intake**					
Energy (kcal/d)	2,141.385 ± 670.8456	2,592.784 ± 696.8506	31.43.792 ± 725.6484	**0.00**	-
Protein (g/d)	67.48 ± 19.00	90.07 ± 21.73	116.48 ± 31.16	**0.00**	**0.00**
Carbohydrate (g/d)	307.93 ± 108.21	377.07 ± 121.67	429.97 ± 111.97	**0.00**	**0.00**
Total fat (g/d)	78.00 ± 31.69	89.47 ± 26.93	116.52 ± 33.07	**0.00**	**0.006**
Vitamin A (RAE)	552.37 ± 305.14	745.43 ± 356.77	992.76 ± 428.89	**0.00**	**0.00**
Vitamin D (μg/day)	1.13 ± 0.82	1.95 ± 1.36	2.82 ± 1.87	**0.00**	**0.00**
Vitamin E (mg/day)	16.37 ± 10.84	16.48 ± 7.95	17.96 ± 7.73	**0.291**	**0.001**
Vitamin K (mg/day)	206.45 ± 264.58	287.64 ± 292.86	375.49 ± 306.99	**0.00**	**0.056**
Thiamin (mg/day)	1.76 ± 0.65	2.12 ± 0.63	2.50 ± 0.73	**0.00**	**0.385**
Riboflavin (mg)	1.68 ± 0.67	2.21 ± 0.59	2.94 ± 0.84	**0.00**	**0.00**
Niacin (mg)	20.38 ± 6.80	25.60 ± 7.11	32.99 ± 11.48	**0.00**	**0.00**
Biotin (μg/day)	28.04 ± 11.62	38.65 ± 12.98	48.30 ± 18.71	**0.00**	**0.00**
Pantothenic acid (mg/day)	4.83 ± 1.48	6.42 ± 1.73	8.09 ± 2.61	**0.00**	**0.00**
Vitamin B6 (mg/day)	1.67 ± 0.53	2.21 ± 0.61	2.70 ± 0.74	**0.00**	**0.00**
Folic acid (μg/day)	581.82 ± 198.92	702.13 ± 224.49	798.57 ± 231.97	**0.00**	**0.301**
Vitamin B12 (μg/day)	2.75 ± 1.04	4.12 ± 1.62	6.19 ± 3.03	**0.00**	**0.00**

CSI, cholesterol–saturated fat index; N6/N3, Σ of Omega 6 series/Σ of Omega 3 series; T, tertile.

Data are presented in the form mean ± SD.

P-value^*b*^: ANCOVA was performed to adjust for a potential confounding factor (energy intake).

p < 0.05 was considered to represent significance.

### Association between the quality of life and its components with N6/N3 ratio and CSI

Raw and adjusted model coefficients and 95% CIs representing associations between QOL (both total and subscale scores) and N6/N3 ratio or CSI tertile are presented in Table 4. Linear logistic regression analysis with adjustment for potential confounders, such as age, energy intake, BMI, employment, and thyroid status, showed that N6/N3 intake ratio was negatively and marginally significantly associated with general health scores (β = −139.94, 95% CI [−286.54, 6.66], *p* = 0.061) and physical role limitations (β= −337.68, 95% CI [−679.99, 1.61], *p* = 0.051). Additionally, in model 1, a significant negative association was observed between N6/N3 intake ratio and social functioning (β = −247.54, 95% CI [−458.14, −36.94], *p* = 0.021), indicating that obese and overweight women consuming a diet with a higher N6/N3 ratio obtained lower scores on social functioning. Finally, there was a significant association between total quality of life score and N6/N3 intake ratio in the raw model (β =64.37, 95% [CI 27.93, 100.82], *p* = 0.001).

**Table 4:** Associations of QOL and its components with N6/N3 intake ratio (*n* = 279) or CSI (*n* = 378) among obese and overweight women.

**Variables^†^**	**N6/N3**				**CSI**			
	**β**	**SE**	**(95% CI)**	***P*** **-value**	**β**	**SE**	**(95% CI)**	***P*** **-value**
**General Health (SF-1)**								
Raw	5.25	10.40	(−15.27, 25.77)	0.614	−0.11	0.18	(−0.47, 0.24)	0.537
Model 1	−139.94	74.30	(−286.54, 6.66)	**0.061**	−0.37	0.22	(−0.47, 0.40)	0.86
**Physical Functioning (SF-2)**								
Raw	3.90	10.42	(−16.66, 24.47)	0.708	0.03	0.18	(−0.38, 0.32)	0.859
Model 1	−88.08	76.34	(−238.71, 62.54)	0.250	−0.70	0.22	(−0.50, 0.36)	0.753
**Physical Role Limitations (SF-3)**								
Raw	9.36	24.01	(−38.00, 56.72)	0.697	0.31	0.39	(−0.46, 1.10)	0.421
Model 1	−337.68	171.97	(−679.99, 1.61)	**0.051**	0.36	0.48	(−0.59, 1.31)	0.455
**Emotional Role Limitations (SF-4)**								
Raw	−32.50	26.84	(−85.44, 20.43)	0.227	−0.00	0.45	(−0.90, 0.90)	0.995
Model 1	−69.29	192.90	(−449.90, 311.31)	0.720	−0.015	0.56	(−1.26, 0.56)	0.782
**Social Functioning (SF-5)**								
Raw	−12.03	14.94	(−41.50, 17.44)	0.422	−0.04	0.24	(−0.52, 0.44)	0.866
Model 1	−247.54	106.74	(−458.14, −36.94)	**0.021**	−0.32	0.30	(−0.091, 0.26)	0.278
**Bodily Pain (SF-6)**								
Raw	−9.59	13.23	(−35.70, 16.51)	0.469	0.24	0.22	(−0.18, 0.68)	0.262
Model 1	−82.93	94.08	(−268.60, 102.63)	0.379	0.43	0.26	(−0.08, 0.96)	0.102
**Vitality (SF-7)**								
Raw	4.27	11.81	(−19.01, 27.56)	0.718	0.09	0.20	(−0.30, 0.48)	0.653
Model 1	−52.44	84.47	(−219.10, 114.21)	0.535	0.24	0.24	(−0.23, 0.72)	0.318
**Mental Health (SF-8)**								
Raw	−11.29	14.27	(−39.64, 17.05)	0.433	0.04	0.24	(−0.043, 0.53)	0.852
Model 1	−85.93	102.16	(−287.50, 115.64)	0.401	−0.06	0.29	(−0.065, 0.52)	0.823
**Health Transition Item (SF-9)**								
Raw	−14.73	17.11	(−48.48, 19.02)	0.390	0.14	0.28	(−0.40, 0.70)	0.601
Model 1	−69.85	123.16	(−312.85, 173.15)	0.571	0.08	0.34	(−0.59, 0.76)	0.806
**Overall QOL (total SF-36 score)**								
Raw	64.37	18.48	(27.93, 100.82)	**0.001**	−0.43	0.41	(−1.25, 0.37)	0.292
Model 1	−1.12	141.21	(−279.74, 277.50)	0.994	0.60	0.52	(0.42, 1.63)	0.247

SE, Standard Error; CI, Confidence Interval; QOL, Quality of Life; SF, Short Form Health Survey; CSI: cholesterol–saturated fat index; N6/N3, Σ of Omega 6 series/Σ of Omega 3 series.

Linear logistic regression was used.

First *P* value is unadjusted (raw) and second *P* value is adjusted for each component.

Adjusted model 1: Adjusted for age, energy intake, BMI, employment, and thyroid status.

## Discussion

We assessed a total of 378 adult overweight and obese women in this cross-sectional study, aiming to elucidate the associations between FAQI and various dimensions of QOL. Our findings revealed that N6/N3 intake ratio was negatively and marginally significantly associated with general health and physical role limitations, after adjustment for potential confounders. Moreover, a significant inverse association between N6/N3 intake ratio and social functioning was observed: specifically, obese and overweight women with a higher N6/N3 intake ratio had lower social functioning scores.

In line with our findings, Ruano et al. have demonstrated a harmful association between intake of fat in the form of saturated and trans fatty acids and several SF-36 domains. In their study, which consisted of 8,430 healthy participants, a significant inverse association was observed between SFA intake and physical functioning as well as general health. Additionally, a significant inverse association was observed between intake of trans unsaturated fatty acids and bodily pain, as well as several mental health domains of QOL, such as social functioning, emotional role limitations, and vitality (19). Furthermore, Lei et al. have reported that dietary patterns involving a higher intake of grains and animal products are associated with poorer functioning in one's roles, while women who consume more fruits and vegetables have better QOL. This finding may be attributed to the ingredients involved in each of these dietary patterns, including refined grains, red and processed meat, and other foods with a high fat content (34). Demark-Wahnefried et al. have reported that, among cancer survivors, a low-fat diet that is high in fruits and vegetables is associated with higher levels of physical functioning. However, they also recommend that patients participate in regular vigorous exercise alongside their diet (35). In agreement with these findings, Ortega et al. have demonstrated that a low-fat, high-fruit/vegetable diet is associated with better physical functioning among elderly Spanish men (36).

In addition to the above findings, Yancy et al. have found that physical functioning, as measured by the SF-36, improves to a greater extent among overweight individuals who follow a 24-week low-fat diet compared to those who follow a low-carbohydrate diet (37). In the same way, a cross-over study among 17 patients with ulcerative colitis (UC) has revealed that a 4-week low-fat diet may be able to improve QOL, as well as decreasing CRP. However, adherence to a low-fat diet can lead to changes in intake of other macronutrients, which may impact the exact results. Moreover, the sample recruited in the aforementioned study consisted of patients with UC, who differ in overall health status from the population of overweight and obese women who participated in our study (26). In addition to these findings, a randomized trial examining the impact of a 12-month weight loss intervention involving either a low-carbohydrate or low-fat diet on QOL among obese patients with type 2 diabetes revealed some associated improvements in QOL. However, the authors also found that changes in dietary macronutrients lead to changes in dietary micronutrients, and the diets investigated were associated with different proportions of protein intake, which could contribute to mood changes (38). Finally, findings from 194 participants in a multicenter randomized clinical trial have suggested that a low-fat (20% of energy intake from fat) and high-fiber diet can improve QOL and increase individuals' confidence in the ability to care for their health (39).

In contrast, several studies have reported that reduced serum cholesterol levels in men may increase the rates of accidental death, homicide, and suicide (40, 41). Furthermore, no changes in QOL were observed among 61 adult patients with type 2 diabetes who adhered to a low-fat diet in a prospective randomized trial (42).

Numerous studies have revealed the potentially harmful effects of trans and saturated fatty acids on risk of coronary heart disease, cardiovascular disease, cognitive decline, Alzheimer's disease, depression, and other chronic diseases (43, 44). Consumption of trans fatty acids may interfere with overall health status *via* several mechanisms, such as interference with neurotransmitter metabolism, inhibition of brain-derived neurotrophic factor expression, and promotion of endothelial dysfunction, systemic inflammation, insulin resistance, and visceral adiposity (45, 46). Additionally, a dietary pattern with a high SFA content produces more inflammation and a less diverse gut microbiome (47). Moreover, dietary changes may lead to weight loss, decreased lipid levels, and increased fitness, which may be associated with scores on the vitality dimension of QOL and with improved physical functioning (48, 49).

The present study is the first to have examined the association between fatty acid quality indices and QOL among overweight and obese women. Moreover, other strengths of the study include the analysis of a large sample and application of multiple adjustments for potential confounders. Several limitations of the present study should also be mentioned. First, the cross-sectional design of the study precludes drawing causal inferences. Additionally, the results are not generalizable to all age groups, to men, or to individuals with particular diseases. Moreover, although the FFQ has been validated for use in the Iranian population, it is not the best method to evaluate intake of certain dietary fatty acids, such as omega-3 PUFAs. Finally, the possibility of recall bias represents a serious limitation, particularly in terms of the assessment of food quality. Thus, it is recommended that these limitations be addressed in future research.

## Conclusion

FAQIs were found to be negatively associated with several measures of QOL in overweight and obese Iranian women. It seems that a low-fat diet with a high intake of fruits and vegetables is linked to improvements in general health, having more energy, and small but significant improvements in physical ability to perform everyday activities (50). Further longitudinal and cohort studies are needed to elucidate the exact association between fatty acid indices and quality of life.

## Data availability statement

The original contributions presented in the study are included in the article/supplementary material, further inquiries can be directed to the corresponding author.

## Ethics statement

The studies involving human participants were reviewed and approved by IR.TUMS.MEDICINE.REC.1401.647. The patients/participants provided their written informed consent to participate in this study.

## Author contributions

NR and KM designed the study and conducted the sampling. NR performed the statistical analyses. NR, ED, NS, CC, and KM wrote the paper. KM takes primary responsibility for the final content. All authors read and approved the final manuscript.
